# Lettuce Cultivar Mediates Both Phyllosphere and Rhizosphere Activity of *Escherichia coli* O157:H7

**DOI:** 10.1371/journal.pone.0033842

**Published:** 2012-03-16

**Authors:** Richard S. Quilliam, A. Prysor Williams, Davey L. Jones

**Affiliations:** School of Environment, Natural Resources and Geography, College of Natural Sciences, Bangor University, Bangor, Gwynedd, United Kingdom; U. S. Salinity Lab, United States of America

## Abstract

Plant roots and leaves can be colonized by human pathogenic bacteria, and accordingly some of the largest outbreaks of foodborne illness have been associated with salad leaves contaminated by *E*. *coli* O157. Integrated disease management strategies often exploit cultivar resistance to provide a level of protection from economically important plant pathogens; however, there is limited evidence of whether the genotype of the plant can also influence the extent of *E*. *coli* O157 colonization. To determine cultivar-specific effects on colonization by *E*. *coli* O157, we used 12 different cultivars of lettuce inoculated with a chromosomally *lux-*marked strain of *E. coli* O157:H7. Lettuce seedlings grown gnotobiotically *in vitro* did exhibit a differential cultivar-specific response to *E*. *coli* O157 colonization, although importantly there was no relationship between metabolic activity (measured as bioluminescence) and cell numbers. Metabolic activity was highest and lowest on the cultivars Vaila-winter gem and Dazzle respectively, and much higher in endophytic and tightly bound cells than in epiphytic and loosely bound cells. The cultivar effect was also evident in the rhizosphere of plants grown in compost, which suggests that cultivar-specific root exudate influences *E. coli* O157 activity. However, the influence of cultivar in the rhizosphere was the opposite to that in the phyllosphere, and the higher number and activity of *E. coli* O157 cells in the rhizosphere may be a consequence of them not being able to gain entry to the plant as effectively. If metabolic activity in the phyllosphere corresponds to a more prepared state of infectivity during human consumption, leaf internalization of *E*. *coli* O157 may pose more of a public health risk than leaf surface contamination alone.

## Introduction

Verocytotoxigenic *Escherichia coli* (VTEC), including *E*. *coli* O157, are commonly isolated from domesticated animals; with the gastrointestinal tract of ruminants, particularly cattle, being widely recognized as the major reservoirs for these human enteric pathogens [1]. However, it is becoming evident that enteric bacteria are also able to survive epiphytically on the surfaces of plants and can successfully colonize plant tissues endophytically [2]. Transmission routes for the contamination of salad-leaf vegetables by zoonotic pathogens include shoot emergence through improperly composted animal wastes together with rain-splash onto the leaves, in addition to irrigation or flooding with contaminated water [3], [4]. The damage caused by insects can also promote internalization of pathogens by providing natural wound openings, while damage by phytopathogens can facilitate colonization of *E*. *coli* O157 by compromising the plant's natural defense mechanisms and sequestering antimicrobial compounds [5]. While the colonization of leaf surfaces is fairly well characterized, the activity of *E*. *coli* O157 on plant roots and in the rhizosphere is still poorly understood, although evidence suggests that entry through roots and endophytic movement throughout the plant can ultimately lead to the internalization of enteric bacteria in leaves [4]. As some of the largest outbreaks of foodborne illnesses have been associated with contaminated salad leaves, fresh fruits and vegetables [6], the carriage and persistence of *E*. *coli* O157 in the rhizosphere is an important public health issue. The colonization of plants by zoonotic enterobacteria, however, is perhaps not surprising as edible plant parts provide an ideal transmission vehicle for re-infection of the primary host. Despite this, relatively little is known about how enteric bacteria grow and survive on their alternate host, although this is currently an active area of research and the subject of several recent reviews [7], [8].

Compatibility between a plant and a plant pathogen involves a complex series of interactions, and while there is a wealth of data on plant-pathogen interactions, there is limited information on how human pathogens interact with their plant hosts. However, it is becoming clear that the colonization of plants by bacterial endophytes is not a passive process and is closely monitored by the plant, which can either enhance or inhibit colonization [9]. Recent evidence suggests that endophytic colonization by *Salmonella* is at least partly regulated by the plant's defense response pathways [10] and genotype [11], [12]. Plants provide a rich and diverse habitat for epiphytic and endophytic microorganisms and all plants, including lettuce, harbor complex communities of microbes [13]. Enteric bacteria colonizing the plant environment therefore have to compete with resident microbial communities that have become highly adapted to this habitat [14]. The aerial plant surface can be a very harsh environment, and is spatially limited by available nutrients and water, together with frequent exposure to sudden changes in temperature and UV radiation. In contrast to the epiphytic niche, the endophytic environment confers a level of protection from abiotic and biotic stresses and contains a plentiful source of nutrients [9].

Integrated disease management strategies for economically important plant diseases routinely include identifying sources of disease resistance through plant breeding and selection. The cultivation of crop varieties with so-called cultivar resistance reduces the need for synthetic pesticides and can provide a fairly long-term form of durable resistance. Lettuce cultivars show varying levels of susceptibility to plant pathogens, e.g. *Fusarium* wilt [15], although it remains unclear whether certain cultivars are more prone to colonization by *E*. *coli* O157. As lettuce is eaten with nominal preparation, the control of plant pathogens by pesticide application in the field, and post-harvest processing for the control of zoonotic pathogens needs to be minimal. Clearly, endophytic colonization of ready-to-eat salad crops by enteric bacteria carries important public health implications, because unlike epiphytic contamination of leaves, internalized bacteria cannot be easily washed off or treated with disinfectants, and may even facilitate passage to the gut. Screening for concurrent resistance to infection by plant pathogens and colonization (or uptake) by zoonotic pathogens would provide a novel approach of plant breeding for cultivar resistance in salad crops.

In this study we aim to quantify the level of metabolic activity and the total number of colony forming units (CFU) of *E. coli* O157 colonizing the phyllosphere and rhizosphere of different cultivars of lettuce. We have tested the hypothesis that plant genotype will affect the level of bacterial colonization, by using a strain of *E*. *coli* O157 carrying a stable chromosomal *lux* reporter (Tn*5 luxCDABE)*. The bioluminescence phenotype of the *lux* biomarker is dependent on the cellular energy status, and as cellular metabolism requires energy, bioluminescence output can be directly related to the metabolic activity of the cells, thus, allowing a quick *in situ* estimation of the size of metabolically active bioluminescent populations [16].

## Materials and Methods

Seeds (Sutton Seeds, Paignton, UK) of 12 different lettuce cultivars (Table 1) were surface sterilized by vigorously shaking in a 10% NaClO solution (containing one drop of Triton X-100) for 20 minutes, followed by several rinses with sterile distilled water. Seeds were then washed in 80% ethanol for a further two minutes and allowed to dry on sterile paper towel. The sterile seeds were then stored for three days in the dark, at 4°C. Following stratification, ten seeds of each cultivar were aseptically sown on the surface of 10% Murashige and Skoog basal medium and 1% agar in Phytatrays (Sigma-Aldrich, Gillingham, UK). Just prior to sowing the seeds, a 1 ml suspension of 10^8^ CFU bioluminescent *E*. *coli* O157:H7 (strain Tn*5 luxCDABE*) [16] from a fresh overnight liquid culture (18 h, 37°C, 150 rev min^−1^, grown in LB broth, washed three times in sterile distilled water and re-suspended in 1 ml of quarter-strength Ringers's solution), was pipetted evenly over the surface of the agar in each tray and allowed to infiltrate for 20 minutes. The sealed trays were transferred to a growth cabinet with a 12 hour photoperiod (irradiance 400 µmol m^−2^ s^−1^; 18°C). At 20 days after sowing (DAS), seedlings were aseptically and carefully removed from the surface of the agar. Each seedling was cut at the base of the hypocotyl, and the root and shoot sections individually assayed for colonization by *E*. *coli* O157. Individual shoot or root sections of each seedling (4 replicate seedlings) were immersed in 1 ml Ringer's solution and vortexed for 1 min. The plant tissue was removed and the wash solution was used to measure loosely attached epiphytic *E*. *coli* O157. The plant tissue (either shoot or root) was then rinsed by vortexing in sterile distilled water three times, and then ground in a 1.5 ml Eppendorf tube for 30 s in 1 ml Ringer's solution with a micro-pestle (Anachem Ltd., Bedfordshire, UK). The resulting homogenate was used to measure endophytic *E. coli* O157 together with those cells that were still firmly attached to the outside of the tissue (referred to as internalized/attached cells). Bioluminescence in the first wash solution and in the ground plant tissue homogenate was immediately measured in plastic luminometer cuvettes with a SystemSURE 18172 luminometer (Hygiena Int., Watford, UK) and expressed as relative light units (RLU). The number of *E*. *coli* O157 cells from these four solutions were simultaneously quantified by plating out 10-fold dilutions and enumerating on plates of cefixime-tellurite sorbitol MacConkey (CT-SMAC) agar (Oxoid Ltd., Basingstoke, UK).

**Table 1:** Lettuce cultivars used in this study.

Cultivar group	Cultivar
Cos	Vaila-winter gem
	Lobjoits green
	Marshall
	Little gem
	Dazzle
Butterhead	Unrivalled
	Rosetta
Crisphead	Lakeland
	Regina dei ghiacci
	Webbs wonderful
	Set (Iceberg)
Loose leaf	Lollo rossa

The effect of lettuce cultivar on *E*. *coli* O157 shoot colonization was further examined by growing two of the cultivars in potting compost. Seeds of the lettuce cultivars Vaila-winter gem and Dazzle were stratified in the dark on damp paper towel at 4°C for 3 days, and sown on moist commercial ‘John Innes Seed’ potting compost (J. Arthur Bower's, Lincoln, UK). A 1.5 ml suspension of approximately 4.7×10^7^ CFU ml^−1^ of *E*. *coli* O157:H7 cells (strain Tn*5 luxCDABE*), was pipetted over each seed and the surrounding compost surface, and the trays transferred to a growth cabinet with the same conditions as above but with a photoperiod of 16 h light and 8 h dark. At 11, 19 and 25 DAS, plants were harvested by excising the shoot at soil level. The shoot was weighed and bioluminescence of *E. coli* O157 cells, and the number of CFU, were simultaneously quantified as described above, except the extraction volume of Ringers solution was 3 ml, and the leaf tissue was ground for 1 minute in a sterile pestle and mortar. The root system was lifted from the soil and lightly shaken, and soil still remaining attached to the roots (the ‘rhizosphere’) was removed and weighed. Sterile Ringers solution was added to rhizosphere soil (3∶1 v/w), vortexed for 1 min and shaken for 15 mins at 225 rev min^−1^ (followed by a further 1 min vortex). The solution was allowed to settle for 5 mins and bioluminescence of *E. coli* O157 cells, and the number of CFU, were quantified from 100 µl aliquots of the supernatant as described above. Persistence of *E. coli* O157 in the soil following the removal of the plant was determined by excising all plants at soil level at 25 DAS and returning the plant-free trays to the growth cabinet. The trays were watered every four days from below and at 28, 38, 58, and 73 DAS rhizosphere soil was taken and bioluminescence and CFU numbers in the soil quantified. At 73 DAS, stratified seeds of the two lettuce cultivars were sown onto the surface on the pots that had previously contained the same cultivar. Bioluminescence and CFU were quantified at 23 DAS (96 days after sowing of the original plants and 71 days after their excision), on the shoot and in the rhizosphere. Measurements of bioluminescence and CFU were analyzed by analysis of variance (ANOVA), Tukey multiple comparison tests, and regression analysis (Minitab 12.0 software, Minitab Inc., PA, USA).

## Results

**Figure 1:**
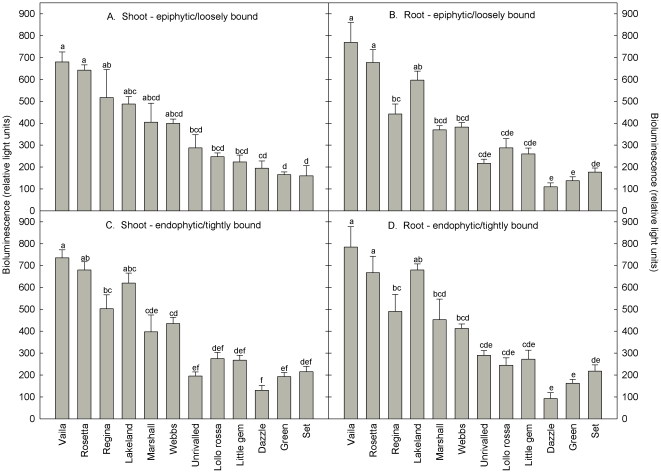
Bioluminescence of *E*. *coli* O157:H7 colonizing the shoot (A and C) and the root (B and D) of different lettuce cultivars. Bars with different letter codes differ significantly from each other (one-way ANOVA, *P*<0.001; Tukey multiple comparison test, *P*<0.01). Data points are the mean of 4 replicates + SEM.

**Figure 2:**
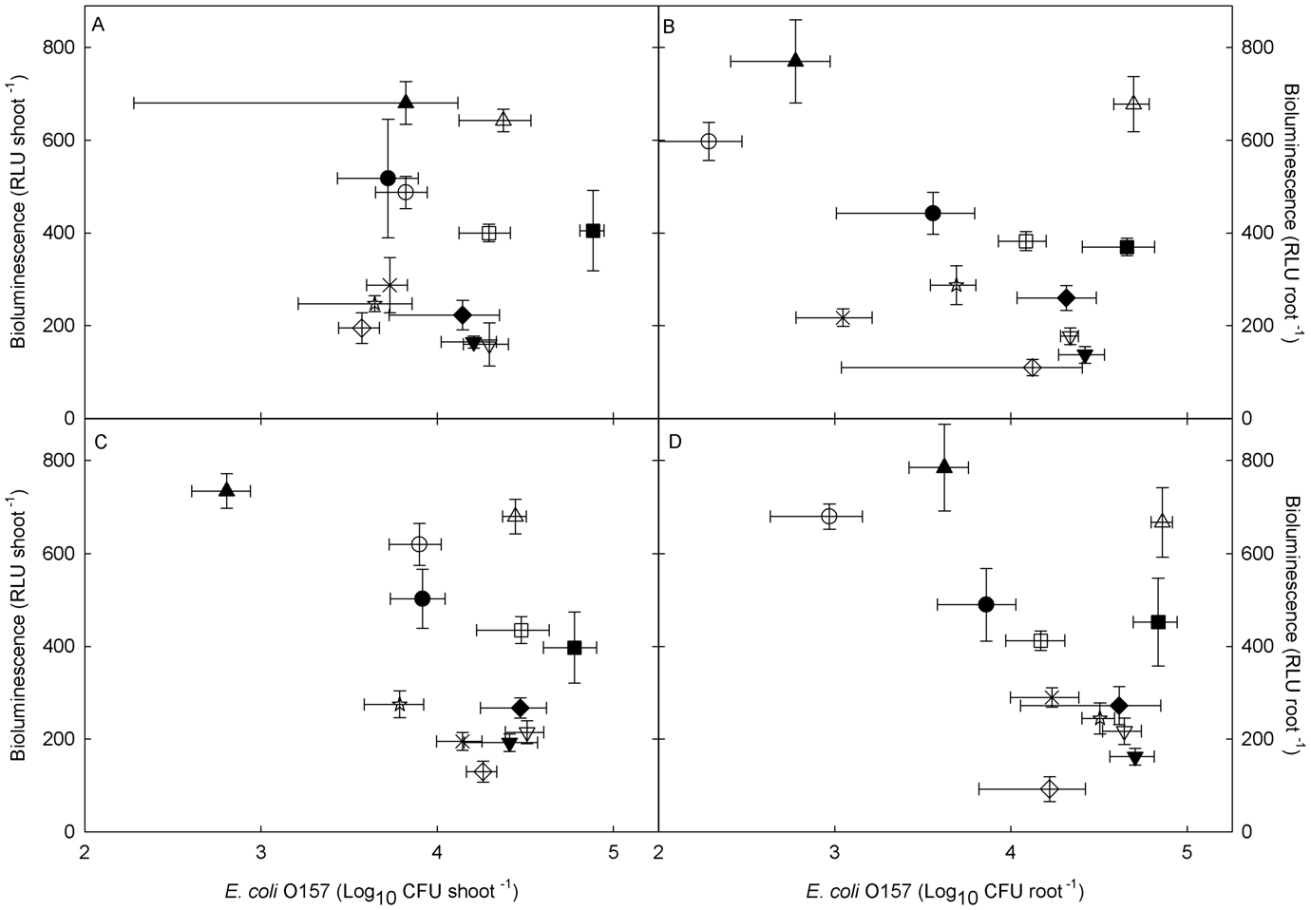
The relationship between CFU and bioluminescence of *E*. *coli* O157:H7 colonizing different cultivars of lettuce. Epiphytic and loosely bound colonization of shoots (A) and roots (B) and colonization by endophytic and tightly bound cells of shoots (C) and roots (D). Lettuce cultivars: Vaila (filled triangle); Rosetta (open triangle); Regina (filled circle); Lakeland (open circle); Marshall (filled square); Webbs (open square); Unrivalled (cross); Lollo Rossa (star); Little Gem (closed diamond); Dazzle (open diamond); Green (filled inverted triangle); Set (open inverted triangle). Data points are the mean of 4 replicates ± SEM.

The bioluminescence of the *lux*-marked *E*. *coli* O157 cells colonizing aseptic lettuce plants varied significantly among the 12 different cultivars tested (Fig. 1). The bioluminescence of epiphytic/loosely bound and internalized/attached cells measured in each individual cultivar was similar, with no significant difference in bioluminescence between the shoot and root tissue. However, when the same cells that had produced the bioluminescence were enumerated on selective media (Fig. 2), there was no significant relationship between the number of CFU and bioluminescence for epiphytic colonization of the shoot or root (R^2^ = 3%, *y* = 366−0.00049*x*, F = 0.1, *P* = 7.59; and R^2^ = 0.05%, *y* = 361−0.00022*x*, F = 0.02, *P* = 0.90 respectively), or internalized/attached colonization of the shoot or root (R^2^ = 6.2%, *y* = 433−0.00262*x*, F = 2.78, *P* = 0.103; and R^2^ = 1.8%, *y* = 423−0.00105, F = 0.77, *P* = 0.385, respectively).

**Figure 3:**
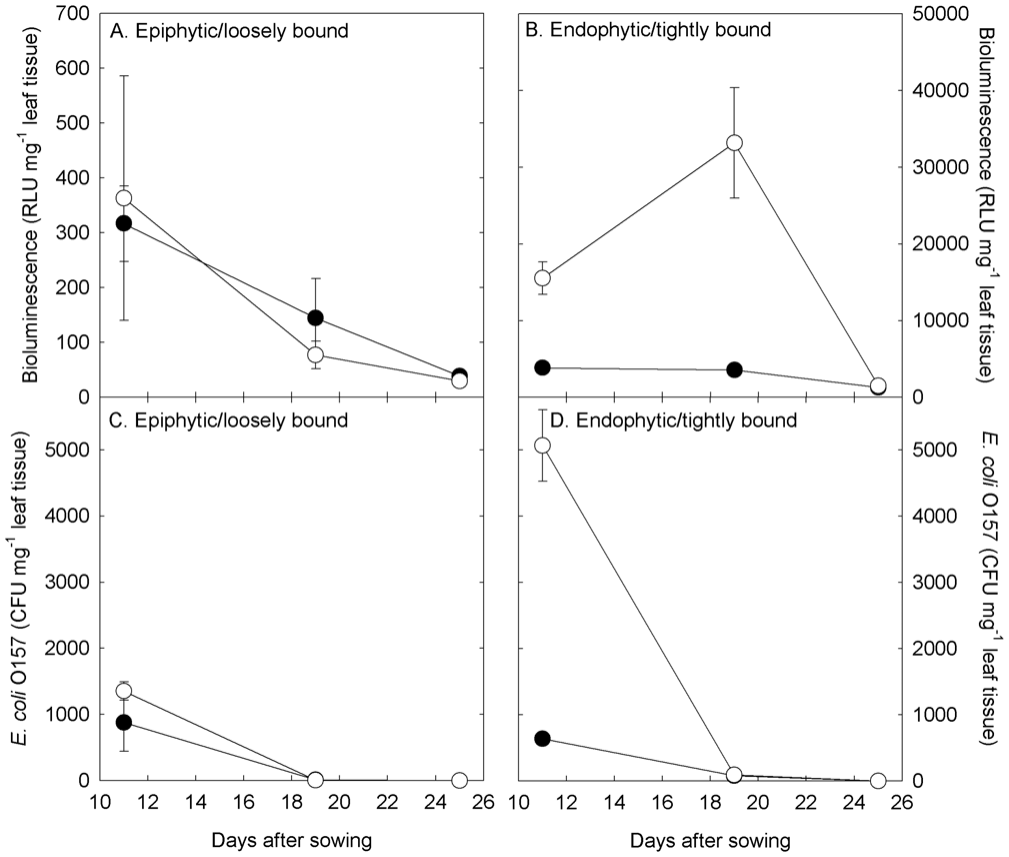
Phyllosphere *E*. *coli* O157:H7 bioluminescence (A and B) and colonisation (C and D) of the lettuce cultivars Vaila (open circle) and Dazzle (filled circle) Data points are the mean of 4 replicates + SEM.

Two contrasting cultivars were selected based on their level of *in vitro* bioluminescence (Fig. 1), i.e. cultivars that had consistently high (Vaila-winter gem) or low (Dazzle) bioluminescence, and were grown in potting compost. Although the bioluminescence and CFU of the epiphytic/loosely bound cells decreased from 11 to 25 DAS, there was no significant difference between the two cultivars of lettuce (Fig. 3). However, at 11 and 19 DAS, the bioluminescence of the endophytic/tightly bound cells colonising the cultivar Vaila was significantly higher than those colonising Dazzle (*P*<0.001). Similarly, at 11 DAS, the number of endophytic/tightly bound CFU was much higher on the cultivar Vaila, although by 19 DAS there was no difference between the two cultivars. For both cultivars the level of bioluminescence was significantly higher in the endophytic/tightly bound cells compared to the epiphytic/loosely bound cells (*P*<0.001).

**Figure 4:**
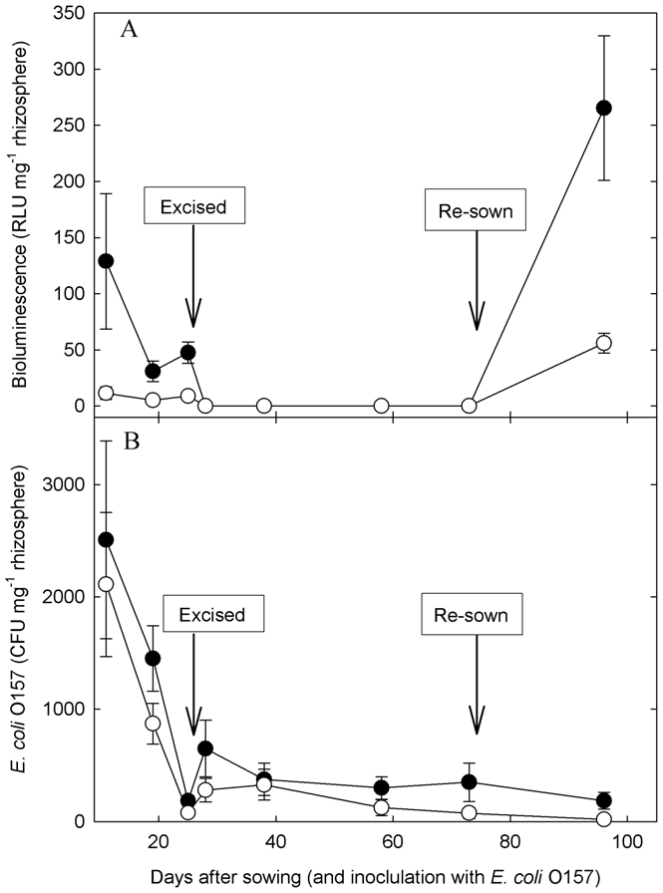
Rhizosphere *E*. *coli* O157:H7 bioluminescence (A) and colonisation (B) of the lettuce cultivars Vaila (open circle) and Dazzle (filled circle). Data points are the mean of 4 replicates + SEM.

In the rhizosphere of the cultivar Dazzle, bioluminescence of *E. coli* O157 was significantly higher than Vaila (*P*<0.001), which was also reflected by the number of CFU (Fig. 4). This is in contrast to the activity of endophytic/tightly bound *E. coli* O157 cells colonising the shoot at 11 and 19 DAS, where the activity of cells on Vaila shoots was significantly higher (Fig. 3). Following excision of the plant however, bioluminescence in the rhizosphere of both cultivars remained below the limits of detection (Fig. 4). The numbers of *E. coli* O157 in the rhizosphere decreased rapidly from 11 to 25 DAS, with slightly higher levels in the rhizosphere of Dazzle. Following excision of the plant, there was a significant increase in *E. coli* O157 numbers in the rhizosphere of both cultivars (*P*<0.05); although from 38 DAS the number of *E. coli* O157 cells remained fairly constant with slightly higher numbers in the rhizosphere of Dazzle.

**Figure 5:**
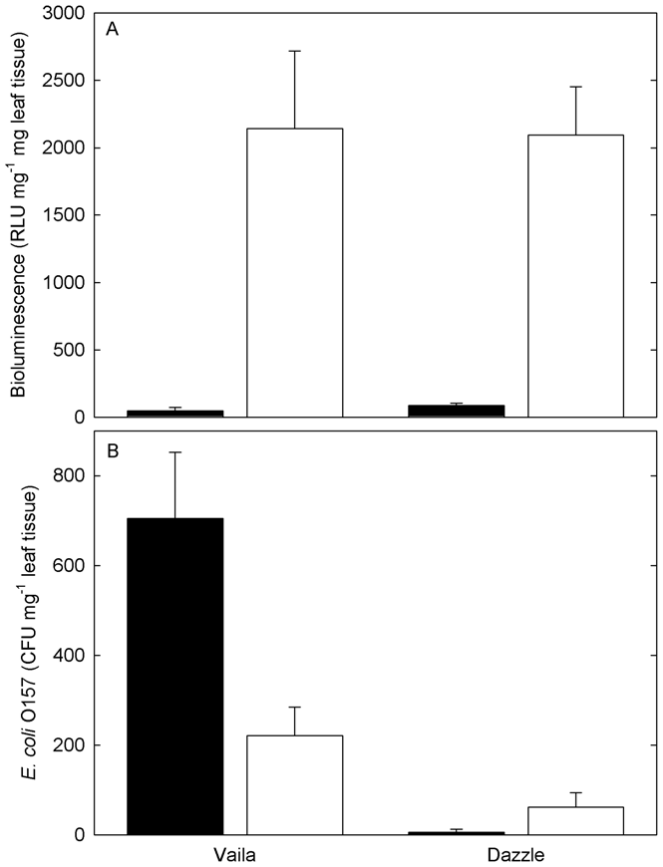
Phyllosphere *E*. *coli* O157:H7 bioluminescence (A) and colonisation (B) of epiphytic and loosely bound cells (black bars) and endophytic and tightly bound cells (white bars) of seedlings 23 days after sowing in soil 96 days after contamination with *E. coli* O157. Data points are the mean of 4 replicates + SEM.

At 73 days after the original sowing (and 48 days after excision), seeds of each cultivar were re-sown into pots that had previously contained the same cultivar. By 23 DAS (96 days since the original inoculation of the soil with *E. coli* O157), there was a significant increase in bioluminescence in the rhizosphere of both cultivars, (despite no increase in CFU), with substantially higher activity in the rhizosphere of Dazzle (Fig. 4). However, the cultivar Vaila had significantly higher numbers of *E. coli* O157 colonising the shoot than Dazzle, with a greater number of epiphytic/loosely bound cells compared to endophytic/tightly bound cells (Fig. 5). There was no significant difference in bioluminescence on the shoot of the two cultivars (Fig. 5), but levels were far lower than they had been on the original plants (Fig. 3 & 5).

## Discussion

The ability of enteric bacteria to actively colonize a number of agriculturally important plants has only fairly recently been recognized as a major public health concern. Previously, contamination of plants by human pathogens such as *E*. *coli* O157 and *Salmonella* was thought to result from passive contamination of leaf surfaces, e.g. through rain splash or from irrigation water. However, a growing number of studies have shown that enteric bacteria possess molecular and physiological mechanisms that allow them to successfully colonize and survive in the plant environment. The internalization of enteric bacteria into plant leaves can occur either directly by entry through wounds or stomata following surface contamination [17], or via root uptake and migration through the vascular system [4]. In this study, we have demonstrated that the potential for *E. coli* O157 colonisation of lettuce is cultivar dependent. Metabolic activity of *E*. *coli* O157 that have become internalized or very firmly attached to the leaf was much greater than those cells on the leaf surface, which suggests that these cells are not only persisting in this environment but are actively metabolizing plant-derived nutrients. Furthermore, the cultivar effect on *E. coli* O157 activity was also evident in the rhizosphere, which may be mediated by cultivar-specific factors such as root exudate [12], root architecture [18] or differences in microbial communities [19]. However, the influence of cultivar on *E. coli* O157 persistence in the rhizosphere was not the same as on the shoot, and the higher number, and activity, of *E. coli* O157 cells in the rhizosphere of Dazzle may be a consequence of them not being able to gain entry to the plant as effectively as Vaila. The metabolic activity of *E. coli* O157 cells in the rhizosphere of both cultivars of lettuce was undetectable following the excision of the host plant and remained undetectable for 48 days; however, re-sowing lettuce seeds triggered the activity of *E. coli* O157 cells in the rhizosphere, and led to the successful colonization of 23 day old plants. This suggests that *E. coli* O157 activity in soil is mediated by plant exudates in the rhizosphere, which is also influenced by the plant cultivar. It is also possible that the increase in bioluminescence in the rhizosphere may, in part, be due to increased metabolic activity of viable but non-culturable (VBNC) *E. coli* O157 cells, which are still capable of colonising plant tissue and producing verotoxins [20].

Lettuce plants exhibit a degree of cultivar resistance to a number of plant pathogens, which is driven by the genotype of the plant, yet resistance is not universal and cultivars bred for resistance to one disease may be more susceptible to another. A recent study reported that the biochemical and morphological characteristics of different lettuce cultivars also significantly influences the structure of bacterial phyllosphere communities [13]. As the characteristics of each lettuce cultivar is a product of the plant's genotype, any level of basal resistance will also be affected by the composition of the microbial community associated with each cultivar. Indigenous microbial communities may also play a significant role in the ability of *E*. *coli* O157 to successfully colonize lettuce seedlings. Evidence for this has been demonstrated by the promotion or inhibition of *E*. *coli* O157 colonization of leaves by epiphytic microbes [2] and provides the basis of novel biocontrol methods for reducing human pathogens on fresh produce [21].

The interaction with other microbes in the rhizosphere will be important for the number of bacterial cells gaining entry to the root and reaching the shoot. Decreased colonization has been observed in lettuce following co-inoculation of seeds with *Enterobacter cloacae*, a species of naturally occurring rhizobacteria [22]. In addition to the activity and composition of indigenous soil microbes, the cultivar of lettuce also greatly influences the metabolic activity and number of *E*. *coli* O157 cells in the rhizosphere. As well as competing with them and grazing on them, soil microbes can also affect signaling molecules in root exudates, which in lettuce has been shown to have a differential cultivar effect on *in vitro Salmonella* chemotaxis [12]. It has been proposed that colonization and internalization by *E*. *coli* O157 is restricted to seedlings, e.g. through entry at root junctions during lateral root development, which may be triggered by the exudates of germinating seeds and developing roots rather than the roots of mature harvestable plants. In addition, recent reports have implied that unnaturally high inoculum levels are needed in order to recover significant levels of *E*. *coli* O157 from harvestable lettuce plants [23], [24]; however, as most studies only quantify colonization by enumerating the number of CFU, the level of colonization could be under-estimated as sub-lethally stressed cells or cells that have entered a VBNC state could remain undetected. The public health significance of this should not be underestimated as post-harvest processing could potentially reactivate dormant cells.

The methods of entry for bacterial endophytes include both direct penetration of the root (e.g. through the production of hydrolytic enzymes) and over the leaf surface (e.g. through stomata), and may occur following shoot emergence through contaminated soil. However, once inside, it is still unclear exactly how bacterial endophytes are transferred around the plant, although it is evident that flagella motility is crucial for both colonisation and epiphytic and endophytic movement of enteric bacteria [2]. Xylem vessels provide the most efficient route for transport from root to shoot, although this niche is nutritionally poor and would not reflect the relatively high metabolic activity of *E. coli* O157 found endophytically colonising lettuce. It is more likely that *E. coli* O157 colonizes the apoplasm and intercellular spaces within parenchyma tissues, which are rich in sugars and non-carbohydrate metabolites [25], [26], while tightly bound *E. coli* O157 cells on the leaf surface could be utilizing localized areas of nutrient exudation.

There is an inconsistency in the literature of reports for root uptake and colonization of lettuce by *E*. *coli* O157, which we believe is due to a combination of host genotype compatibility with pathogen serovar, the developmental stage of the leaf sampled, autochthonous rhizosphere and phyllosphere microbial communities, environmental variables and the methods used for quantifying colonization. Most studies on the interactions between plants and human pathogenic bacteria have used the enumeration of bacterial cells (as CFU) to determine the extent of colonization. While this method provides a proxy for quantifying the level of contamination, it assumes that every cell enumerated is in a metabolically active state and represents an equal chance of causing human infection following ingestion. The measurement of luminescence from a *lux-*reporter construct allows the real-time culture-independent quantification of the level of metabolic activity, which has previously been used as an estimation of pathogen infectivity [27]. We have shown that the level of activity is not correlated with the number of CFU (which could be due to the age of the individual cell, and the heterogeneous nature of the plant tissue environment, e.g. accessibility and the localized exhaustion of nutrients and water, and the localized competition with each other and other microbes), and while the numbers of epiphytic and internalized/attached *E*. *coli* O157 cells colonizing lettuce plants growing in compost are similar, the activity of the endophytic/tightly bound *E*. *coli* O157 cells is much higher. Increased metabolic activity may result from the sudden flux of nutrients released from the plant homogenate; however, the time between grinding the plant tissue and measuring the bioluminescence was less than 70 seconds, which is insufficient for any significant increase in luminescence by *E. coli* O157 (pers. comm. Graeme Paton, unpublished data). Clearly, further work is needed to establish whether human pathogens epiphytically colonizing leaf tissue pose as much of a public health risk as those internalized in the leaf tissue, by determining whether the increased metabolic activity of endophytic pathogens is linked to the increased likelihood of human infection.
